# Cigarette smoking is an independent risk factor for post-stroke delirium

**DOI:** 10.1186/s12883-017-0840-3

**Published:** 2017-03-23

**Authors:** Tae Sung Lim, Jin Soo Lee, Jung Han Yoon, So Young Moon, In Soo Joo, Kyoon Huh, Ji Man Hong

**Affiliations:** Department of Neurology, School of Medicine, Ajou University, 5 San, Woncheon-dong, Yongtong-gu, Suwon-si, Kyunggi-do 442-749 Republic of Korea

**Keywords:** Post-stroke delirium, Ischemic stroke, Acute stroke, Smoking

## Abstract

**Background:**

Post-stroke delirium is a common problem in the care of stroke patients, and is associated with longer hospitalization, high short-term mortality, and an increased need for long-term care. Although post-stroke delirium occurs in approximately 10 ~ 30% of patients, little is known about the risk factors for post-stroke delirium in patients who experience acute stroke.

**Methods:**

A total of 576 consecutive patients who experienced ischemic stroke (mean age, 65.2 years; range, 23–93 years) were screened for delirium over a 2-year period in an acute stroke care unit of a tertiary referral hospital. We screened for delirium using the Confusion Assessment Method. Once delirium was suspected, we evaluated the symptoms using the Korean Version of the Delirium Rating Scale-Revised-98. Neurological deficits were assessed using the National Institutes of Health Stroke Scale at admission and discharge, and functional ability was assessed using the Barthel Index and modified Rankin Scale at discharge and 3 months after discharge.

**Results:**

Thirty-eight (6.7%) patients with stroke developed delirium during admission to the acute stroke care unit. Patients with delirium were significantly older (70.6 vs*.* 64.9 years of age, *P* = .001) and smoked cigarettes more frequently (40% vs*.* 24%, *P* = .033) than patients without delirium. In terms of clinical features, the delirium group experienced a significantly higher rate of major hemispheric stroke (55% vs*.* 26%, *P* < .001), exhibited poorer functional performance at discharge and 3 months after discharge, and stayed in hospital significantly longer. Independent risk factors for delirium were older age, history of cigarette smoking, and major hemispheric stroke.

**Conclusion:**

Abrupt cessation of cigarette smoking may be a risk factor for post-stroke delirium in ischemic stroke patients. The development of delirium after stroke is associated with worse outcome and longer hospitalization.

## Background

Delirium is an acute confusional state characterized by decreased attention, an altered level of consciousness, and decreased cognitive function including impairment of memory and thought processes [1]. Substantial numbers of delirium cases are caused by systemic inflammation due to an infection or surgery in patients with predisposing risk factors, such as an old age, head trauma, stroke, or underlying dementia [2]. Delirium occurs in 10 ~ 48% of patients during admission and results in poorer functional outcome(s), lower quality of life, and longer hospitalization [3, 4]. Although the exact pathophysiology of delirium is not well understood, decreased acetylcholine and increased dopamine levels are believed to be important factors in this process [5].

Ischemic stroke is caused by an occlusion of the cerebral arteries, which may lead to neurological deficit. Presently, admission to a dedicated stroke care unit is the standard treatment for acute stroke care, and is associated with a long-term benefit in neurological and functional outcomes [6]. However, admission to such an unfamiliar environment for stroke patients and an abrupt cessation of cigarette and alcohol use could induce delirium [7]. Few studies, however, have investigated these variables as risk factors for post-stroke delirium.

The present study evaluated retrospective data from ischemic stroke patients in an acute stroke care unit over a 2-year period to identify the risk factors for post-stroke delirium.

## Methods

### Subjects and delirium screening

We screened a total of 589 consecutive ischemic stroke patients who were admitted to an acute stroke unit over a 2-year period. Of these patients, 13 were transferred out of the unit within 48 h and, thus, were excluded from further study, yielding a total of 576 patients who were enrolled in the present retrospective study. All patients were evaluated using brain computed tomography (CT) and magnetic resonance imaging (MRI); 28 patients showed negative results after brain magnetic resonance diffusion-weighted imaging.

Delirium was screened daily during admission using the Confusion Assessment Method (CAM) [8]. The diagnosis of delirium was based on the following four features: (i) acute onset and a fluctuating course; (ii) inattention; (iii) disorganized thinking; (iv) an altered level of consciousness. Once delirium was suspected, we evaluated the symptoms using the Korean Version of the Delirium Rating Scale-Revised-98 (K-DRS-R-98), which quantifies multiple parameters, such as the sleep-wake cycle, perception, hallucinations, delusions, mood, language, thought process, psychomotor behavior, orientation, attention, imprinting, short-term memory, and visuospatial orientation [9]. All items were scored on a 4-point scale, resulting in a total score range of 0 to 39.

### Definition of variables

We divided all stroke patients into 5 groups based on the modified Oxfordshire Community Stroke Project criteria, which included major hemispheric, minor hemispheric (subcortical), lacunar, brainstem/cerebellar, and non-classified syndromes [10]. We recorded right, left, and bilateral hemispheric involvement. Cigarette smoking was defined as current smoking of any number of cigarettes. Daily alcohol use was defined as consuming more than 3 standard drinks per day. Infection during admission to the stroke unit was defined as a body temperature >38.5 °C [11]. Metabolic derangement was defined as follows: sodium <130 or >150 mmol/L; glucose <2.8 or >14 mmol/L; an estimated glomerular filtration rate <30 mL/min; calcium <2 or >2.75 mmol/L; and oxygen saturation <90% [11]. Neurological deficits were assessed using the National Institutes of Health Stroke Scale (NIHSS) at admission and discharge, and functional ability was assessed using the Barthel Index (BI) and modified Rankin Scale (mRS) at discharge and 3 months after discharge. Follow-up data at 3 months after discharge were available for 414 patients.

### Statistics

The Student’s *t*-test and chi-square test were used to compare differences between patients with and without delirium. Logistic regression analysis was performed with delirium occurrence as the dependent variable to analyze independent risk factors and calculate odds ratios (OR). All statistical analyses were performed using SPSS version 18.0 (SPSS Inc., Chicago, IL, USA).

## Results

**Fig. 1:**
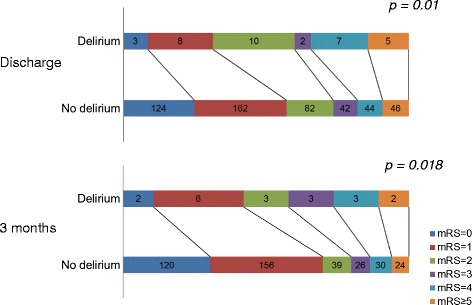
Modified Rankin Scale (mRS) at discharge and 3 months after discharge

A total of 576 ischemic stroke patients were investigated. The characteristics of these patients are summarized in Table 1. Among them, 368 patients (63.9%) were male and 25% (*n* = 144) were cigarette smokers. The average NIHSS at admission was 5.5 (range 0–29). The comparison between patients with and without post-stroke delirium is shown in Table 2. Thirty-eight of 576 patients (6.7%) developed delirium during admission to the acute stroke care unit. Compared with patients without delirium, patients with delirium were significantly older (70.6 vs*.* 64.9 years of age, *P* = .001) and smoked cigarettes more frequently (40% vs*.* 24%, *P* = .033). In terms of clinical features, the delirium group had a significantly higher rate of major hemispheric stroke (55% vs*.* 26%, *P* < .001), and showed a worse modified Rankin score at discharge (2.5 vs*.* 1.7, *P* = 0.01) and at 3 months after discharge (2.3 vs*.* 1.4, *P* = .018) (Fig. 1). The length of hospitalization was longer in patients with delirium (13.3 days vs*.* 10.7 days, *P* = .049). The univariate and multivariate logistic regression analyses are shown in Table 3. In the multivariate analysis of risk factors for delirium, independent risk factors for delirium were older age (OR = 1.05), cigarette smoking (OR = 2.8), and major hemispheric stroke (OR = 4.8) after adjustment for age, sex and alcohol use.

**Table 1:** Characteristics of the 576 patients with ischemic stroke

Characteristics	Values
Age, y, mean (range)	65.2 (23–93)
Male sex (%)	368 (63.9)
Hypertension (%)	382 (66.3)
Diabetes (%)	210 (36.5)
Cigarette smoking (%)	144 (25)
Daily alcohol use (%)	35 (6.1)
Underlying dementia (%)	11 (1.9)
NIHSS at admission, mean (range)	5.5 (0–29)
Delirium (%)	38 (6.7)

*NIHSS* National Institutes of Health Stroke Scale

**Table 2:** Comparison between patients with and without post-stroke delirium

	Delirium (*n* = 38)	No delirium (*n* = 538)	*p*-value
Demographics			
Age (SD), y	70.6 (9.3)	64.9 (13.4)	0.001*
Male sex (%)	28 (73)	339 (63)	0.409
Hypertension (%)	29 (76)	353 (65)	0.215
Diabetes (%)	18 (47)	192 (36)	0.164
Smoking (%)	15 (40)	129 (24)	0.049*
Daily alcohol use (%)	5 (13)	30 (5)	0.072
Underlying dementia (%)	0 (0)	11 (2)	1.000
Clinical features			
Involved hemisphere			0.751
Right (%)	16 (42)	194 (36)	
Left (%)	16 (42)	268 (49)	
Bilateral (%)	5 (13)	49 (9)	
Stroke syndrome			<0.001*
Major hemispheric (%)	21 (55)	140 (26)	
Minor hemispheric (%)	3 (8)	76 (14)	
Lacunar syndrome (%)	5 (13)	169 (31)	
Brainstem-cerebellum (%)	2 (5)	84 (16)	
Infection during admission (%)	2 (5)	18 (3)	0.637
Metabolic derangement (%)	8 (21)	87 (16)	0.433
NIHSS at discharge (SD)	5.0 (5.0)	3.5 (5.9)	0.142
BI at discharge (SD)	73.8 (29.0)	83.2 (29.0)	0.065
mRS at discharge (SD)	2.5 (1.6)	1.7 (1.6)	0.010*
Inpatient mortality (%)	0 (0)	4 (1)	1.000
Hospitalization period (SD), d	13.3 (7.0)	10.7 (8.0)	0.049*
BI 3 months after discharge (SD)	79.0 (34.5)	89.5 (25.0)	0.247
mRS 3 months after discharge (SD)	2.3 (1.6)	1.4 (1.5)	0.018*

*NIHSS* National Institutes of Health Stroke Scale, *BI* Barthel index, *mRS* modified Rankin scale, * p-value < 0.﻿05

**Table 3:** Multivariate logistic regression analysis of risk factors for delirium

	Univariate analysis		Multivariate analysis	
	*p*-value	OR (95% CI)	*p*-value	OR (95% CI)
Age	0.011*	1.038 (1.008 ~ 1.068)	0.004*	1.048 (1.015 ~ 1.081)
Male sex	0.197	1.631 (0.776 ~ 3.428)	0.194	1.748 (0.753 ~ 4.060)
Hypertension	0.182	1.689 (0.783 ~ 3.643)		
Diabetes	0.151	1.622 (0.838 ~ 3.140)		
Cigarette smoking	0.036*	2.068 (1.048 ~ 4.081)	0.015*	2.764 (1.215 ~ 6.289)
Daily alcohol use	0.068	2.566 (0.934 ~ 7.045)	0.345	1.722 (0.558 ~ 5.317)
Right hemispheric involvement	0.377	1.381 (0.674 ~ 2.830)		
Stroke syndrome (ref. lacunar syndrome)				
Major hemispheric	0.001*	5.070 (1.864 ~ 13.791)	0.003*	4.781 (1.711 ~ 13.356)
Minor hemispheric	0.698	1.334 (0.311 ~ 5.726)	0.809	1.199 (0.275 ~ 5.221)
Brainstem-cerebellum	0.227	1.988 (0.652 ~ 6.063)	0.294	1.831 (0.592 ~ 5.664)

* p-valu e < 0. 05

## Discussion

To our knowledge, the present study was the first to identify cigarette smoking as a risk factor for post-stroke delirium. Our 2-year retrospective study suggests that older age, history of cigarette smoking, and major hemispheric stroke are independent risk factors for post-stroke delirium, which is associated with a poorer functional outcome and longer hospitalization.

Cigarette smoking was an independent risk factor for post-stroke delirium after adjustment for age, sex, and alcohol use. Although the exact mechanism of the contribution of cigarette smoking to delirium has yet to be elucidated, abrupt cessation of smoking and withdrawal of nicotinic stimulation has been postulated [7]. A recent systematic review suggested that current research evidence is insufficient to determine whether cigarette smoking is a risk factor for delirium [12]. However, there have been no systematic reviews to investigate post-stroke delirium cases with a relatively higher incidence of smoking. In contrast to cigarette smoking, alcohol consumption was not associated with delirium. This finding was consistent with previous studies that also reported no relationship [13, 14]. In clinical practice, alcohol withdrawal syndromes are usually considered in the management of patients with stroke, but cigarette withdrawal is not. Further studies are needed to ascertain the relationship between cigarette smoking, its proper management, such as the nicotine patch, and post-stroke delirium.

The incidence of post-stroke delirium in the present study was 6.7%, which was lower than that reported in previous studies [4]. This lower incidence is possibly due to the younger age of the stroke cohort in the present study. The mean age of the patients in the present study was 65.2 years, which is more than 7 years younger than that reported in recently published studies [11, 13]. The lower incidence of delirium in this young study cohort corresponds well with a study that had a mean age of 57.3, and reported a delirium incidence of 13%, which was significantly lower than other studies that were conducted at that time [14]. Younger age also affected the lower incidence of dementia in this population, as we could not find a significant association between preexisting dementia and post-stroke delirium. Another explanation for the lower incidence of delirium is our inclusion of patients who experienced only ischemic stroke. Previous studies report that post-stroke delirium is more frequent in hemorrhagic stroke than ischemic stroke [14, 15].

Our results suggest that major hemispheric stroke was another independent risk factor for post-stroke delirium. This finding is also consistent with previous studies [13, 16, 17]. However, other clinical factors, such as a high initial NIHSS and right hemispheric stroke, were not identified in the present study. These variables were associated with delirium in some studies [13, 14], but not in other studies [15, 18]. In previous studies, cortical involvement and subsequent decreased cortical function, such as neglect syndrome or aphasia, were important in the pathogenesis of post-stroke delirium [14, 16, 19, 20]. Our study cohort had a higher proportion of patients who experienced lacunar stroke and, in this particular population, the high NIHSS was not the result of cortical dysfunction, but motor-sensory dysfunction, which was not associated with delirium.

Patients with delirium exhibited poorer functional outcome at discharge and experienced longer hospitalization. In addition, the long-term outcome at 3 months after discharge was worse in the delirium group. These findings are consistent with the results of many previous studies [4]. Compared with our study, a recent review and meta-analysis evaluating the outcomes of acute stroke patients with delirium reported higher inpatient mortality (OR = 4.7) and a hospital stay that was 9 days longer [4]. We did not report high inpatient mortality because the overall mortality rate was very low (0.7%) due to the number of patients with a low stroke severity. Longitudinal follow-up of these patients regarding their long-term mortality and morbidity that includes their post-stroke dementia status is needed to elucidate the long-term effect of delirium in young patients who experience low severity stroke.

The present study had limitations stemming from its retrospective design. First, pre-stroke cognition was not measured using a structured questionnaire. Instead, we included the patient history of dementia diagnosis for analysis. Second, anticholinergic medication(s) was not recorded, and the use of this medication could be associated with delirium [17]. Furthermore, a prospective studies involving a structured questionnaire to assess pre-stroke cognition and medication use, and treatment trials involving cholinesterase inhibitors [21, 22] are warranted.

## Conclusions

A history of cigarette smoking before stroke may be a risk factor for post-stroke delirium, most likely due to abrupt cessation of smoking, especially in young patients who experience a low severity stroke. The development of delirium after stroke was associated with a worse outcome and longer hospitalization.

## Abbreviations

BI: Barthel index
CAM: Confusion assessment method
CT: Computed tomography
K-DRS-R-98: Korean version of the delirium rating scale-revised-98
MRI: Magnetic resonance imaging
mRS: Modified rankin scale
NIHSS: National institutes of health stroke scale
